# A long way to syndromic short stature

**DOI:** 10.1186/s13052-024-01737-3

**Published:** 2024-09-27

**Authors:** Federica Gaudioso, Camilla Meossi, Lidia Pezzani, Federico Grilli, Rosamaria Silipigni, Silvia Russo, Maura Masciadri, Alessandro Vimercati, Paola Giovanna Marchisio, Maria Francesca Bedeschi, Donatella Milani

**Affiliations:** 1 Clinical Genetics Unit, Fondazione IRCCS Ca’ Granda Ospedale Maggiore Policlinico, Milano, 20122 Italy; 2 Paediatric Unit, ASST Papa Giovanni XXIII, Bergamo, 24127 Italy; 3 Laboratory of Medical Genetics, Fondazione IRCCS Ca’ Granda Ospedale Maggiore Policlinico, Milano, Italy; 4 Experimental Research Laboratory of Medical Cytogenetics and Molecular Genetics, IRCCS Istituto Auxologico Italiano, Via Ariosto 13, Milano, 20145 Italy; 5 Fondazione IRCCS Ca’ Granda Ospedale Maggiore Policlinico, Milano, 20122 Italy; 6 Department of Pathophysiology and Transplantation, University of Milan, Milano, Italy; 7 Unità di Genetica medica, Fondazione IRCCS Ca’ Granda, Ospedale Maggiore Policlinico, Via della Commenda, 9, Milano, 20122 Italy; 8 Department of Developmental Neuroscience, IRCCS Stella Maris Foundation, Pisa, Italy

**Keywords:** Case report, Short stature, Silver Russell Syndrome, Temple syndrome, *NSD1* duplication

## Abstract

**Background:**

Silver-Russell Syndrome (SRS, MIM #180860) is a clinically and genetically heterogeneous disorder characterized by intrauterine and postnatal growth retardation; SRS is also accompanied by dysmorphic features such as triangular facial appearance, broad forehead, body asymmetry and significant feeding difficulties. The incidence is unknown but estimated at 1:30,000-100,000 live births. The diagnosis of SRS is guided by specific criteria described in the Netchine–Harbison clinical scoring system (NH-CSS).

**Case presentation:**

Hereby we describe four patients with syndromic short stature in whom, despite fitting the criteria for SRS genetic analysis (and one on them even meeting the clinical criteria for SRS), molecular analysis actually diagnosed a different syndrome. Some additional features such as hypotonia, microcephaly, developmental delay and/or intellectual disability, and family history of growth failure, were actually discordant with SRS in our cohort.

**Conclusions:**

The clinical resemblance of other short stature syndromes with SRS poses a risk of diagnostic failure, in particular when clinical SRS only criteria are met, allowing SRS diagnosis in the absence of a positive result of a genetic test. The presence of additional features atypical for SRS diagnosis becomes a red flag for a more extensive and thorough analysis. The signs relevant to the differential diagnosis should be valued as much as possible since a correct diagnosis of these patients is the only way to provide the appropriate care pathway, a thorough genetic counselling, prognosis definition, follow up setting, appropriate monitoring and care of possible medical problems.

## Background

Short stature is usually defined as a height of at least 2 standard deviations (SD) less than the mean of a specific population. This definition includes 2.3% of the population and usually includes healthy individuals. On the contrary, SD below 2.5 or 3 (which would comprise approximately 0.6 and 0.1% of the population, respectively) is frequently associated with syndromic conditions and usually caused by a monogenic defect. Syndromic short statures are a wide group of pathologies that includes more than 1,000 conditions, the most exemplifying being Silver Russell Syndrome (SRS, MIM #180860) [1, 2].

SRS is a distinct syndromic growth disorder with prenatal and postnatal growth failure and an incidence of 1 per every 30,000 to 100,000 live births. An International Consensus in 2017 summarized the recommendations for clinical and molecular diagnosis and management of SRS [1–3].

This consensus suggested adopting the Netchine-Harbison clinical scoring system (NH-CSS) for SRS. NH-CSS has the following six key features: (1) small for gestational age (SGA), (2) postnatal growth failure, (3) relative macrocephaly at birth, (4) protruding forehead, (5) body asymmetry, and (6) feeding difficulties and/or low body mass index. Patients with four or more NH-CSS criteria are defined as “clinical SRS”. The threshold for molecular testing (≥ 3 of six criteria) is lower than that needed for a clinical diagnosis of SRS (≥ 4 of six criteria)) [1, 2].

The most frequent epigenetic causes of SRS are loss of methylation on chromosome 11p15 (11p15 LOM) (50%) and maternal uniparental disomy for chromosome 7 (upd(7)mat) (10%).

If testing of both 11p15 LOM and upd(7)mat is negative, additional molecular testing can be considered. There are a small number of individuals with SRS who have duplications, deletions or translocations involving the imprinting centres at 11p15.5 or chromosome 7. There are also rare descriptions of affected individuals with pathogenic variants in *CDKN1C*,* IGF2*,* PLAG1*, and *HMGA2* [2].

However, following NH-CSS, about 40% of clinical SRS remains undiagnosed, highlighting the need to define the molecular aetiology in a consistent fraction of unsolved patients [4–7].

We describe four further patients referred to the Paediatric Genetic Unit of “Fondazione IRCCS Ospedale Maggiore” of Milan, Italy, with the suspicion of SRS, in whom, after a careful clinical evaluation and an accurate medical history assessment, other genetic diagnoses were confirmed.

Based on these cases and on data from the literature, we aim to deepen the knowledge on less frequent SRS-like differential diagnoses.

Our aim is not only to extend the pool of SRS-like pathologies but also to highlight the criticity and the challenge of the molecular investigation, in particular deepening the role of the relative macrocephaly, that represents a crucial diagnostic decision crossroad.

## Case presentation

### Patient 1

The first patient is a female who came to our attention aged 2 years. SRS was suspected due to SGA, poor postnatal growth, failure to thrive and hypotonia.

She is the fourth child of unrelated parents, coming from different regions in Morocco. Her family history was negative for inherited diseases. Prenatal infectious screenings were unremarkable, but a prenatal ultrasonography showed intra-uterine growth restriction (IUGR). She was delivered at 35 + 3 weeks of gestation age by caesarean section because of spontaneous onset of labour.

At birth, weight was 1622 g (-2.07 SDS), length 43 cm (-1.37 SD), and head circumference 30 cm (− 1 SD). APGAR score at the first and fifth minute of life were 7 and 8. She developed transient hypoglycaemia, treated with an intravenous glucose infusion.

**Fig. 1:**
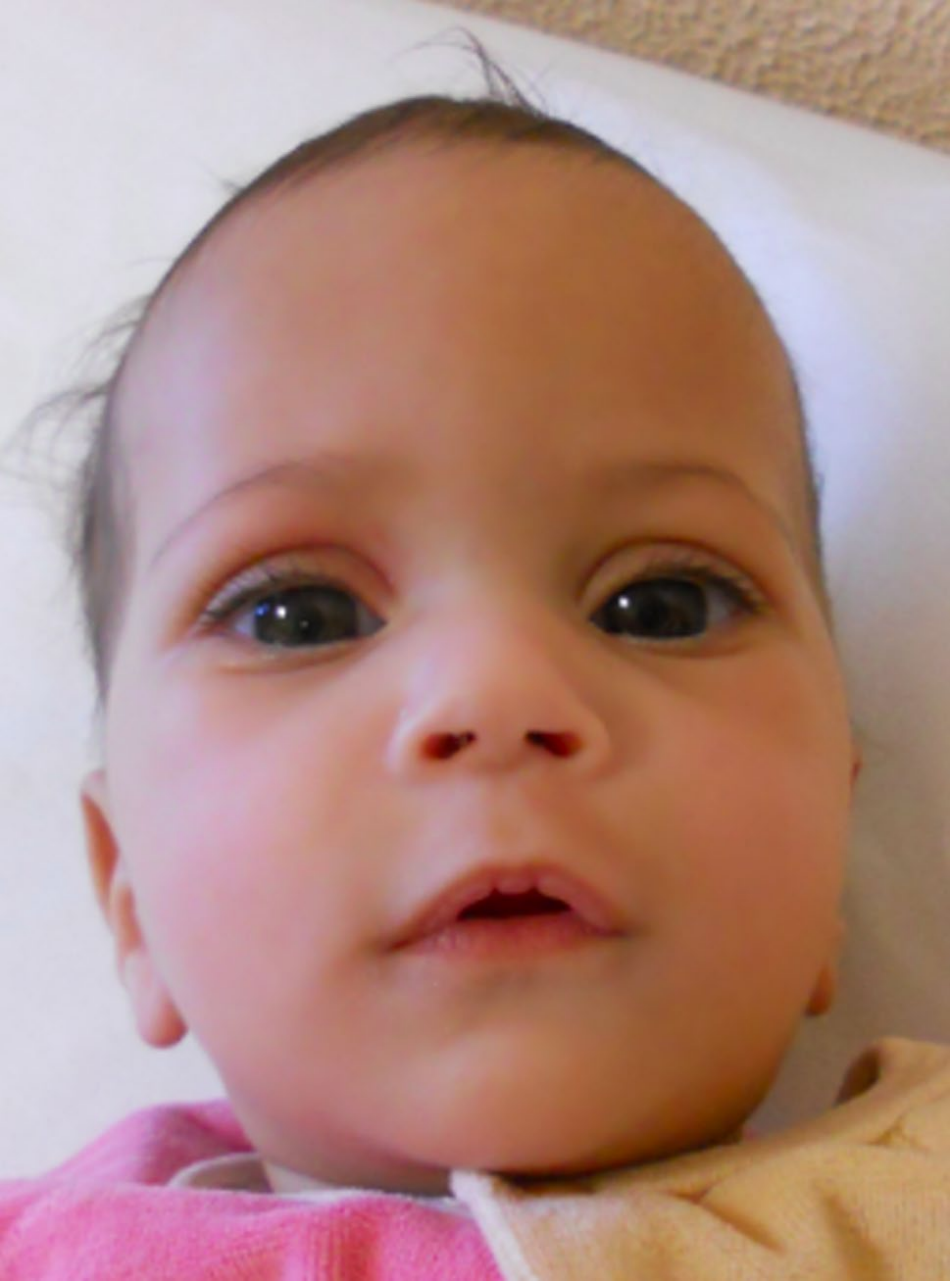
Clinical photograph of patient 1, frontal view: *note* bitemporal constriction, saddle nose with long philtrum and a prominent upper lip

**Fig. 2:**
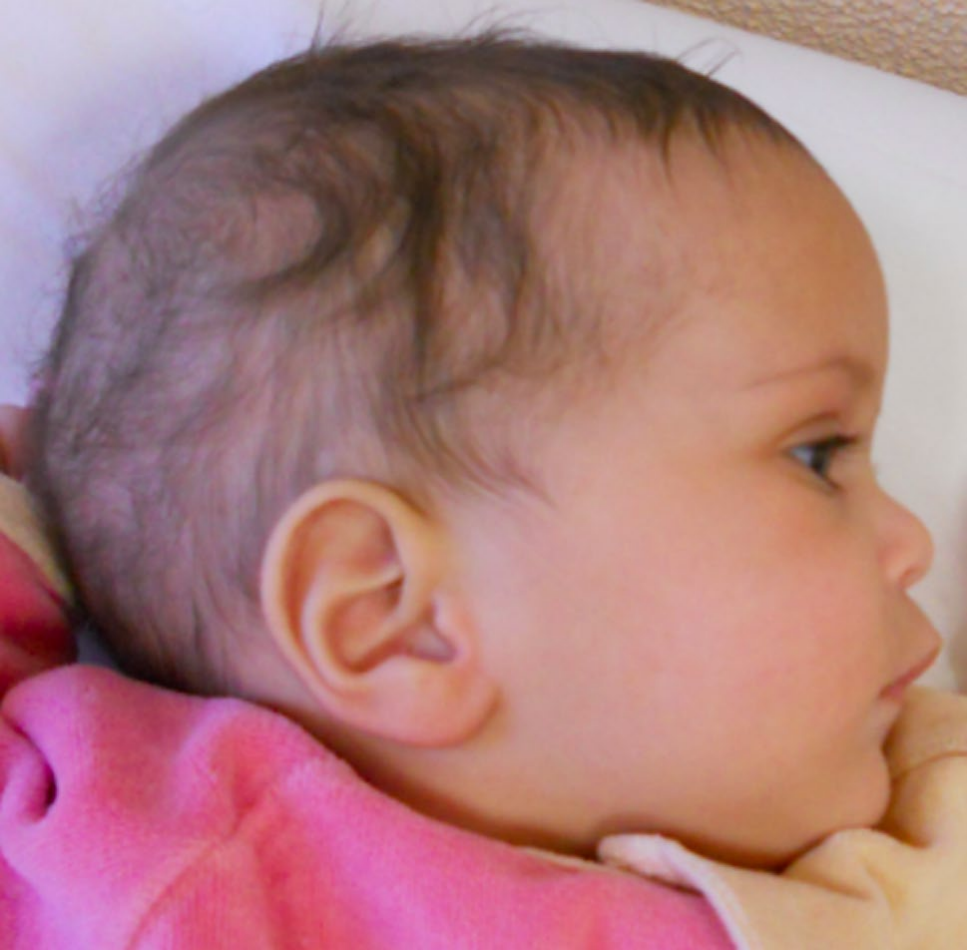
Clinical photograph of patient 1, lateral view: *note* dolichocephaly, a prominent forehead, micrognathia

At the time of examination, she showed dolichocephaly, a prominent forehead, bitemporal constriction, saddle nose with long philtrum, a prominent upper lip, micrognathia and ogival palate (Figs. 1 and 2). Delayed motor development and hypotonia were present. Blood tests were normal except for a mild elevation of aminotransferase, with normal creatinine kinase. Echocardiogram and ophthalmological evaluation were normal. Brain magnetic resonance imaging (MRI) showed mild hypoplasia of vermis and corpus callosum. She weighed 6.4 Kg (-7.5 SD) and was 75.4 cm (-2.75 SD) long.

At 3 years of age, she weighed 7.5 Kg (-7.1 SD) and was 81.3 cm (-3.3 SD) long.

At 4 years and 7 months of age she showed language difficulties and motor delay. Her growth values were height 93.5 cm (-2.5 SD), weight 10.5 kg (-4.8 SD), body mass index (BMI) 12 Kg/m3 (-3 SD), growth velocity 5.4 cm/year (-1.1 SD), with mild bone age retardation (4 years).

Poor growth was associated with low levels of IGF-1, and she began therapy with recombinant Growth Hormone (GH) aged 5, with good clinical response. At the age of 6, she developed early puberty and was ok treated with GnRH analogue. She developed mild hypermetropia and strabismus over time, and her muscular strength and tone markedly improved.

Minor dysmorphic findings, severe growth retardation and marked muscular hypotonia prompted genetic analysis seeking an underlying genetic cause.

Genetic workup ruled out Wolf Hirschhorn syndrome, Fluorescent in situ hybridization [(FISH) 4p16.3] and Prader-Willi syndrome [FISH 15q11.2] and the methylation studies were both normal). Her karyotype was normal (46, XX).

Therefore, a Array -Comparative Genomic Hybridization (CGH-Array) was performed, which showed a de novo 1 Mb deletion of the chromosome 14 in the q32.2-32.31 imprinted region arr [hg19] 14q32.2-32.31 (100449043–101488936)x1 A microsatellite segregation analysis showed maternal only microsatellites in the deleted region, establishing that the deletion was on the paternal chromosome 14, and resulting in a diagnosis of Temple syndrome (TS14).TS14 by microdeletion have been described and reported in literature [8].

### Patient 2

The second patient is an Asian male who came to our attention aged 1 year and 3 months with short stature and feeding difficulties since birth. He was born via caesarean section without complications at 38 + 1 weeks of gestation after a spontaneous pregnancy with a birth weight of 2230 g (-2.38 SD), a length of 44 cm (-2.65 SD), and a head circumference of 33,1 cm (-1 SD). Since the first days of life, he showed failure to thrive.

**Fig. 3:**
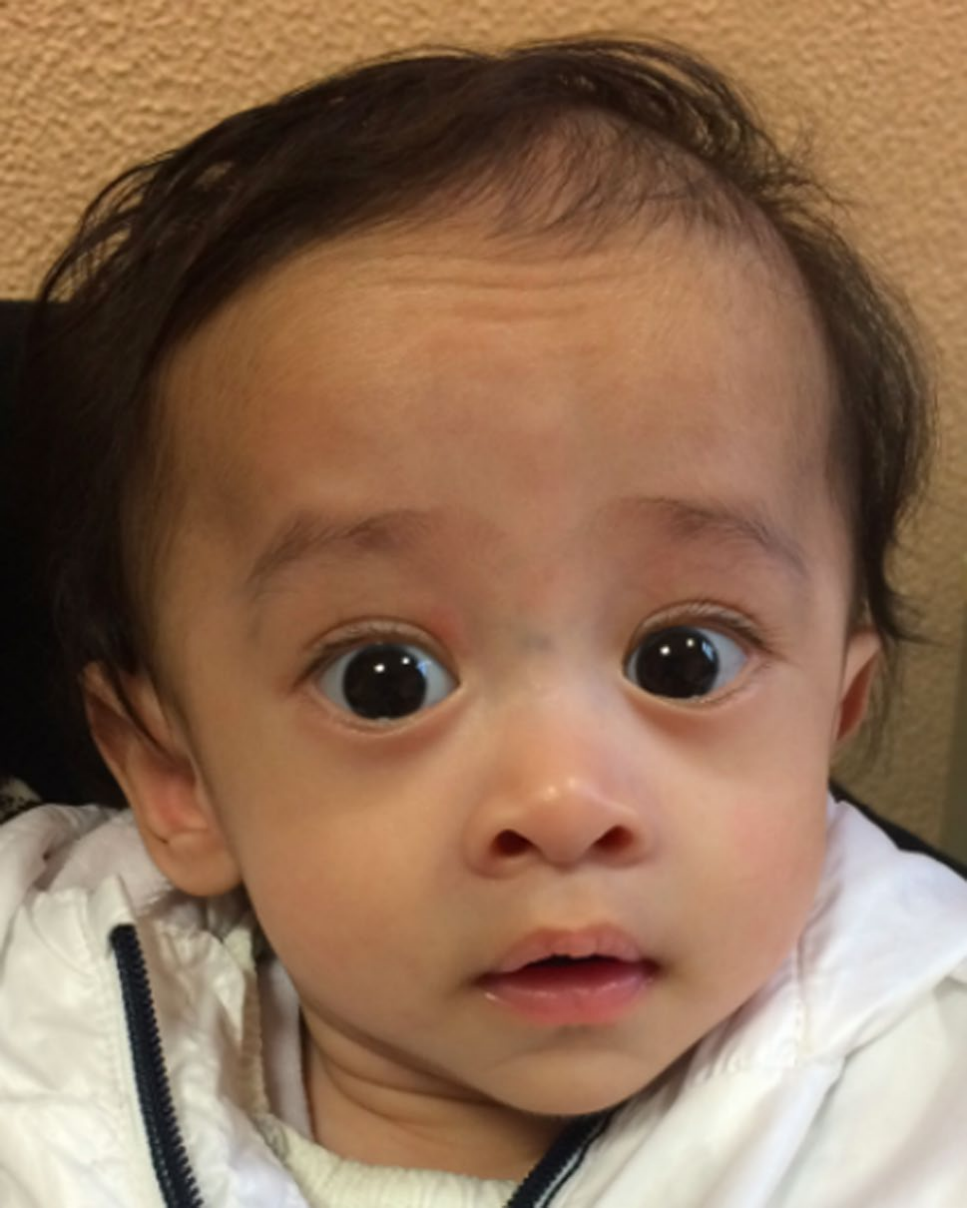
Clinical photograph of patient 2, frontal view: *note* triangular face characterized by a broad, slightly rounded and protruding forehead, slight epicanthus, a small angioma of the nasal root, short philtrum, pointed chin

**Fig. 4:**
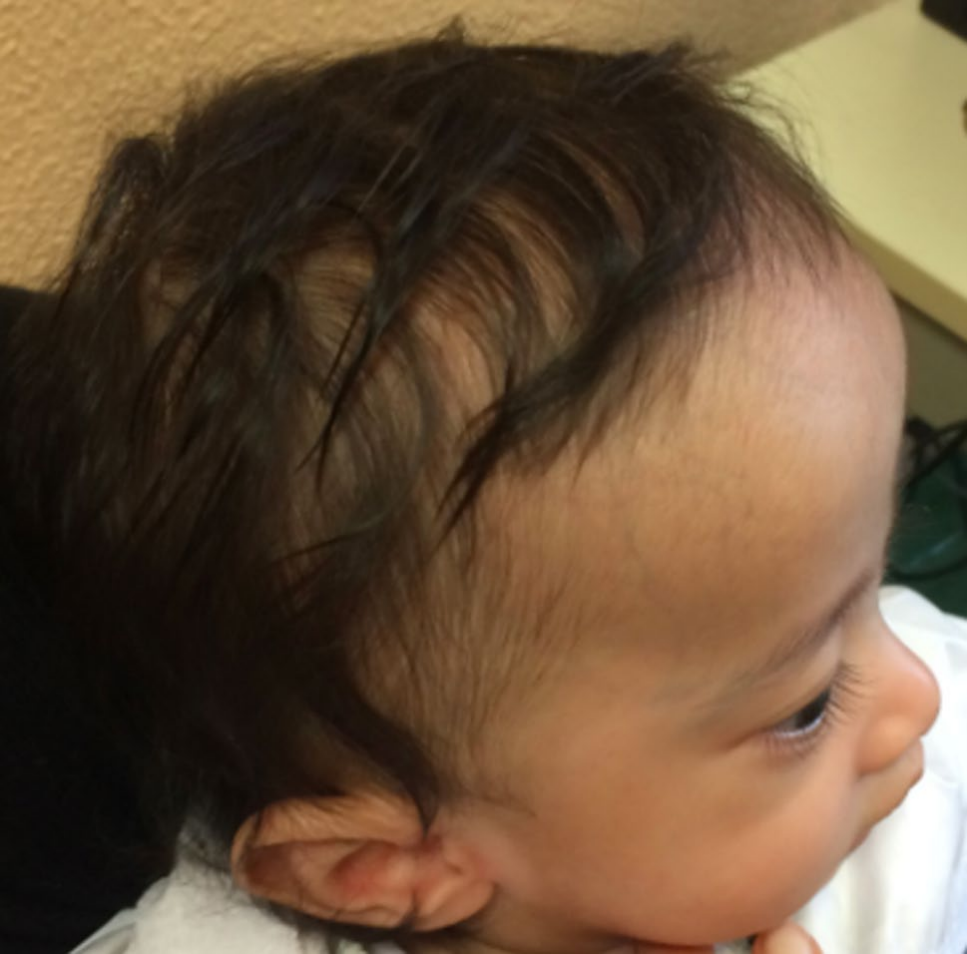
Clinical photograph of patient 2, lateral view: *note* a prominent forehead and low-set ears

At 5 months, at first genetic evaluation, facial dysmorphisms were noted, including a slightly triangular face characterized by a broad, slightly rounded and protruding forehead, slight epicanthus, a small angioma of the nasal root, short philtrum, pointed chin and low-set ears (Figs. 3 and 4). He also had sternal bone spurs and mild diastasis of the rectus abdominis muscles, a Mongolian patch on the left ankle and in the sacral region, and fifth finger clinodactyly, without any skeletal asymmetry. Screening for metabolic disease was negative (urinary and blood amino acids and urinary organic acids). At the time of evaluation his weight was 4.150 g (-5 SD), his length 60 cm (-2.7 SD), and head circumference 38.5 cm (-3.5 SD).

At 1 years and 3 months his growth was still harmonic with a weight of 7600 g (-2.7 SD), a length of 73.5 cm (-2 SD) and head circumference of 43.2 cm (-2.7 SD).

A karyotype and a molecular analysis for SRS (chromosome 7 and chromosome 14 UPD research, a CGH Array and IC1 methylation analysis) were performed with normal results.

Because the clinical phenotype was highly consistent with SRS, further genetic testing to investigate possible SRS-like syndromes was performed, showing a maternal uniparental disomy of the entire Chromosome 20 (UPD20), an imprinting disorder known as Mulchandani-Bhoj-Conlin Syndrome (OMIM# 617352) [9].

### Patient 3

The third patient is an Italian male referred aged 19 months. SRS was suspected because of postnatal growth defect and mild craniofacial dysmorphisms. He also manifested language delay, failure to thrive, and atopic eczema. He was the youngest of five siblings. His family history was positive for a growth defect in his brother. Gestation and neonatal periods were regular. He was born at 38 weeks of gestational age with a birth weight of 2550 g (-1.5 SD), a length of 47 cm (-1,5 SD) and a head circumference of 32 cm (-2 SD). At the time of examination, motor development was consistent with his age, but he showed mild expressive language delay (he could only say one word). Parents report feeding difficulties during his postnatal period. At 19 months, he showed severely impaired growth with a weight of 9,6 Kg (-1.5 SD), a height 74 cm (-3 SD), a head circumference of 44 cm (-2 SD). On clinical examination, he was observed to have a wide nasal root, a hint of epicanthus on the left, elongated eyelashes and microretrognathia.

At 4 years and 5 months of age his weight was 12,5 Kg (-2.5 SD), his height was 93 cm (-3 SD), his BMI was 14,5 (-1.5 SD) and his head circumference was 46 cm (-3 SD). He underwent genetic analysis because of the suspicion of a syndromic short stature condition. CGH array showed a microduplication of 1.2 Mb in the 5q35.2q35.3 region (arr[hg19] 5q35.2q35.3(175839681–177047120)x3, encompassing the *NSD1* gene. Both his brother affected by growth delay and his mother tested positive for the same microduplication, assessing the same diagnosis of *NSD1* duplication-associated syndromic short stature in both brothers and likely in the mother.

*NSD1* duplication are associated with duplication-related SRS-like 5q35 condition. The clinic of his brother was consistent with poor growth. At birth his weight was 2650 g (-1 SD), his length was 47 cm (-1.5SD), and his CC was 34,5 cm (-0.3 SD). at the time of the evaluation he was 12 years old. His weight was 29 Kg (-1.5 SD), his height was 132 cm (-2 SD), and his head circumference was 48 cm (-3 SD). About his facial characteristics he has synophrys, reverse epicanthus, and thin vermillions.

The mother of the children has a harmonic short stature. She referred that she needed a scholar support during her infancy. Unfortunately we didn’t have the possibility to reach more informations, but looking at the hereditary model and the high penetrance of this condition we could make the same diagnosis in all the three family members.

After the diagnosis, a more accurate neurodevelopmental evaluation was done at the age of 30 months, showing an IQ of 71 points on a Griffiths scale. An echocardiography ruled out congenital heart diseases.

### Patient 4

The fourth patient was referred aged 14 months and he was the only male child born from healthy non-consanguineous parents admitted to the genetic paediatric department of our Institute due to short stature.

His gestation was characterized by IUGR and polyhydramnios and was born via an induced delivery at 38 weeks of gestational age.

His birth weight was 2530 g (-1.5 SD), length 48 cm (-1 SD) and head circumference 34 cm (-0.4 SD). His poor growth was confirmed also during his postnatal life.

His physical examination evidenced triangular face, prominent forehead, depressed (deeply set) eyes, bulbous nose, micrognathia, short philtrum and thin vermillion. He had an apparently inhomogeneous distribution of subcutaneous adipose tissue, concentrated in the upper body, especially arms, becoming more evident with increasing age. His weight was 7100 g (− 3 SD), length 72 cm (− 2.5 SD), head circumference 44,6 cm (-1.5 SD). A cardiac ultrasound revealed a small patent foramen ovale. Electrocardiogram (ECG), abdominal ultrasound and transfontanellar ultrasound were normal.

A CGH array study showed a de novo 300 kb microdeletion at 18q21.32, of uncertain significance.

Analysis of 11p15.5 ICR1/ICR2 and maternal UPD7 studies showed a normal methylation pattern. Due to the high suspicion of SRS or SR-like syndromes, Next generation sequencing analysis (NGS) of *IGF2*,* CDKN1C*,* PLAG1*,* PIK3RI*,* HMGA2* and other genes associated with the clinical characteristics of the patient was performed, revealing a point pathogenetic heterozygous mutation on the *PIK3RI* gene (NM_181523.3:c.1945 C > T, p.Arg649Trp), pathogenetic for SHORT syndrome.

## Discussion and conclusions

All patients in our cohort fulfill the criteria for molecular testing as recommended by NH-CSS [1, 2] (Table 1); they are referred to the Paediatric Genetic Unit of “Fondazione IRCCS Ospedale Maggiore”, Milan (Italy) with suspected SRS, but were diagnosed with different conditions.

**Table 1:**
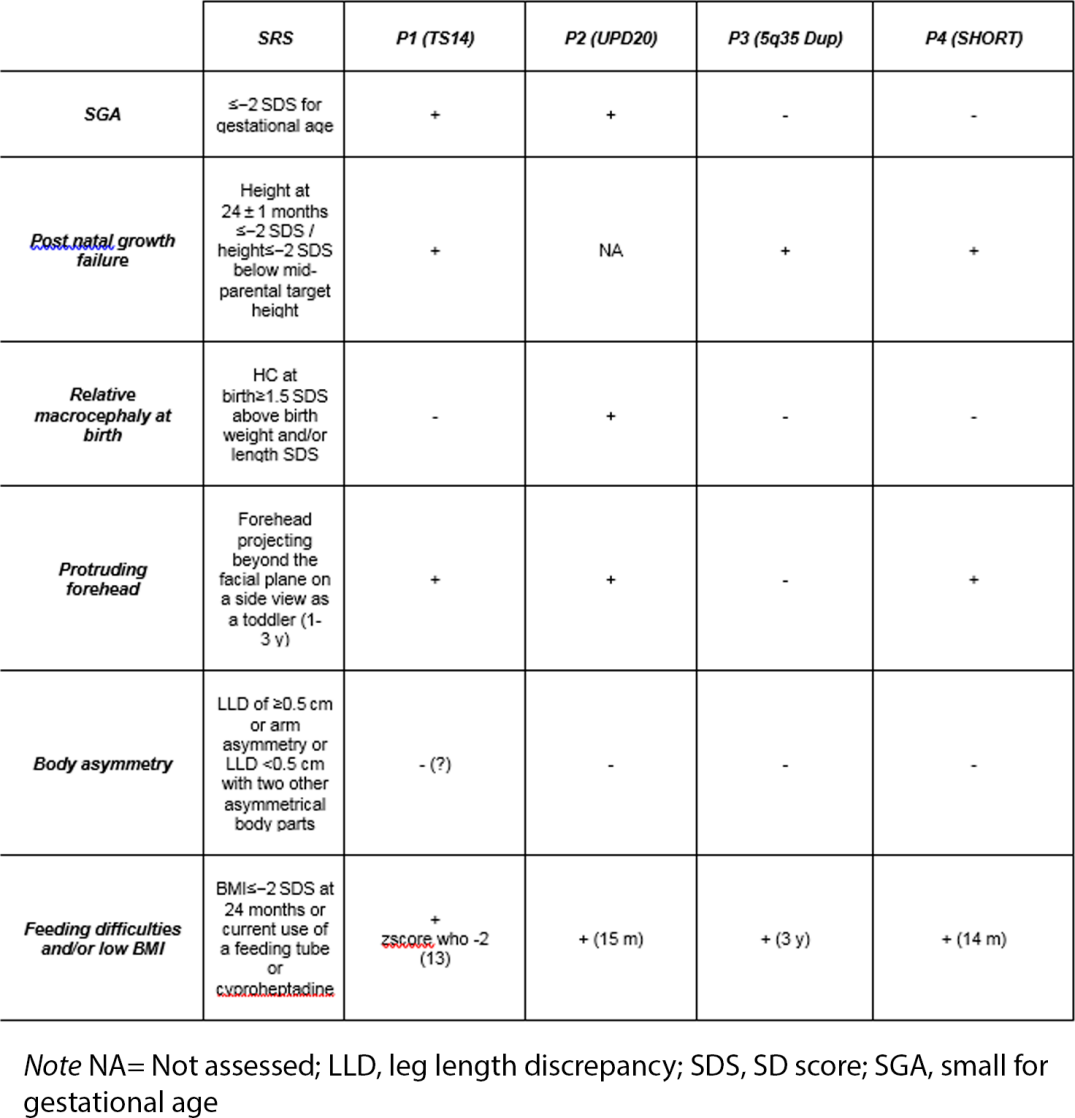
Comparison between Netchine-Harbison clinical scoring system and clinical featuresof our cases

None of them actually presented with asymmetry, and all showed distinctive features which might suggest a differential diagnosis. In particular, the first patient had severe hypotonia, the second growth delay without relative macrocephaly, the third a strong positive family history for short stature with psychomotor delay and microcephaly, and the fourth a lipodystrophy.

Our first patient was diagnosed with TS14 (OMIM #616222), characterized by IUGR and post-natal failure to thrive, relative macrocephaly, developmental delay, hypotonia, poor feeding, body asymmetries, ligamentous hyperlaxity, scoliosis, GH deficiency and precocious puberty. The literature reports a small subset of TS14 with features apparently overlapping with SRS; however, no patient satisfied all of the six NH-CSS criteria. Muscular hypotonia is more severe in TS14 than in SRS, whereas body asymmetry and feeding difficulties are less typical of TS14 and more in keeping with SRS [1, 8, 10–12].

According to Kagami et al., genetic testing for TS14 should be considered in patients with pre- and postnatal growth failure with the coexistence of PWS-like marked hypotonia and SRS-like relative macrocephaly, prominent forehead, and feeding difficulty in infancy [8].

In the second patient a diagnosis of UPD(20)mat (OMIM #617352) was confirmed. This syndrome is characterised by severe feeding difficulties associated with failure to thrive, preterm birth, and intrauterine/postnatal growth retardation [9, 13]. To the best of our knowledge, it has been described in less than 30 patients. Neither macrocephaly nor asymmetry, which are typical of SRS, are found in our second patient, in line with the existing literature. We suggest that, in the absence of macrocephaly and/or asymmetry, UPD(20)mat should be considered before carrying out SRS-specific tests. A more comprehensive single nucleotide polymorphism (SNP) array test or a wider methylation test, including multiple clinical DMRscould be performed as first tier when the patient’s features are in keeping with both SRS and UPD20 [9, 13].

In the third patient CGH array revealed a 5q35 duplication, a copy number variation (CNV) already described in literature but not specifically reported in differential diagnosis with SRS, adding a further to the heterogeneous group of SRS-associated chromosomal imbalances. These patients show growth retardation with several dysmorphic features. However, the major difference between typical SRS and 5q35 duplication is that the latter patients are microcephalic and show mild developmental delay [14–16]. The last patient had a mutation in *PIK3RI*, causing SHORT Syndrome (OMIM #269880), characterised by Short stature, Hyperextensibility of joints or hernia (inguinal) or both, Ocular depression, Rieger anomaly, and Teething delay. In particular, the dysmorphic features are similar to SRS and include triangular facies, and lack of facial fat. Like in SRS, developmental milestones and cognition are normal for individuals with SHORT syndrome. The literature reports two patients with features apparently overlapping with those of SRS [17]. Because SRS and SHORT may be clinically undistinguishable, the 2016 Consensus advises to include SHORT syndrome in the differential diagnoses of SRS [2].

In summary, our findings of pathogenic variants in genes or genomic regions associated with differential diagnoses of SRS reflect the molecular heterogeneity in patients referred for SRS testing.

We suggest that further investigations for alternative diagnoses should be consideredin patients without all six NH-CSS items: attention should be addressed to additional features atypical for SRS such as hypotonia, microcephaly, developmental delay and/or intellectual disability, and family history of growth failure [3, 17, 18]. Despite fulfilling SRS criteria, and if methylation analysis consistent with the main clinical suspicion are negative, we suggest using SNP array in the presence of discordant features, particularly if there are developmental delay, intellectual disability and/or some malformation such as congenital heart diseases and genitourinary anomalies. Actually, more conditions related to UPD, not only 14 and 20, but also 6, 15, and 16, have been included in the differential diagnosis of SRS, as well CNVs in several chromosomes [3, 17, 18]. In these patients SNP array could give a good implementation in terms of diagnostic rate because it detect copy number variants as well uniparental disomy. If SNP array is negative, we should proceed with NGS. Multiple conditions identifiable via whole exome sequencing (WES) can be differential diagnoses of SRS. Specifically, besides SHORT diagnosed in patient 4, we need to consider the syndromes already suggested by the 2017 Consensus and confirmed by recent studies (i.e. 3-M syndrome, Mulibrey nanism, Floating harbour syndrome etc.) [4–7]. Early and specific diagnosis is important for individualised management and to optimise growth and long-term outcomes. Genomic diagnosis allows physicians to refer patients to appropriate specialists, offer disease-specific follow-up and detailed preventive information to families according to the different situations [4–7].

In each of the described conditions there are some more specific features that need a well-timed management, thus highlighting to the importance of a correct molecular diagnosis.

A consistent part of these patients’ management of course refers to the short stature, but mild or no modifications of GH or IGF-1 levels are reported in some syndromic conditions with short stature, and response to GH treatment, if given, varies depending on the underlying syndromic diagnosis. A GH supplementation may affect growth velocity, thus suggesting that the pathway is growth hormone responsive, but not all syndromic short statures are GH-responsive [19]. This again leads to another reason for ensuring appropriate molecular diagnosis in patients with clinical SRS. GH provocation tests in TS14 shows apparent GH deficiency (GHD) in 2 of 13 patients examined by Kagami et al. 2017 and Brightman et al. 2019 report that a short-term GH supplementation improved the height of 7 out of 14 patients with TS14 [8, 20]. Patients with UPD(20)mat benefit from GH therapy. Two UPD(20)mat patients reported by Tannorella et al., 2021 had GH deficiency and reported significant growth acceleration after GH treatment, in keeping with the research reported by Mulchandani et al., 2016. One UPD(20) patient was diagnosed with scoliosis during the GH supplementation without central hypotonia or other predisposing factors for scoliosis, thus showing the need for monitoring of this potential adverse event in patients with UPD(20) [9, 13]. GH in 5q35 microduplication has not been well reported in the literature to the best of our knowledge [14–16]. Indeed, we only found one report by Bernhardt et al., 2021 describing the GH status of a 2-year-old girl with a normal GH secretion. Supplementation was assessed and this patient’s response was at least as good or better than the typical patient with growth hormone deficiency despite being growth hormone sufficient, suggesting that the *NSD1* pathway is growth hormone responsive [19]. Patients with SHORT syndrome need a different management. GH treatment could aggravate pre-existing insulin resistance and accelerate the onset of diabetes mellitus as noted by Avila et al., 2016. Therefore, its use in patients with SHORT syndrome should be evaluated with caution [17].

Compared to SRS, each syndrome has some additional peculiar clinical aspects that are important to know as soon as possible for the correct management (Table 2). TS14 patients may be affected by precocious puberty; therefore, GnRH treatment and monitoring of bone age by radiological assessment is recommended because the advanced bone age of patients has a negative impact on adult height and the efficacy of GH treatment [12].

**Table 2:**
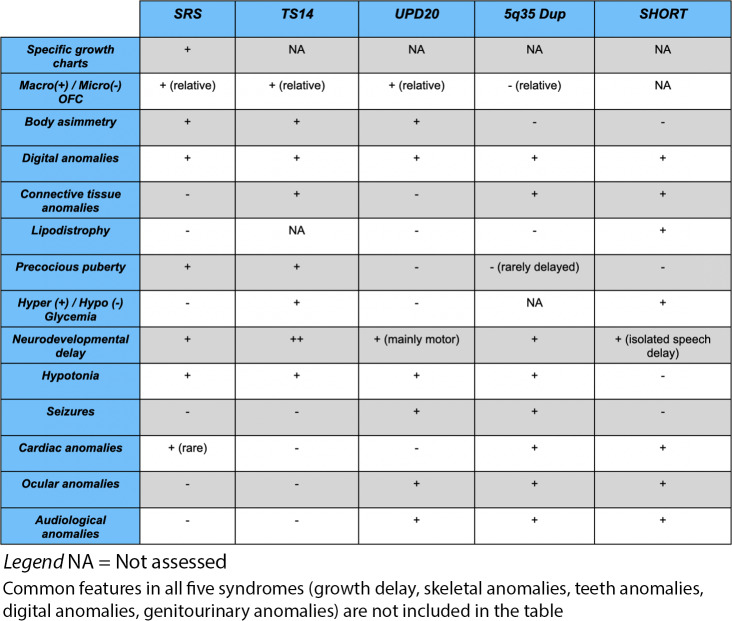
Summary of different manifestations in short stature syndromes discussed in this paper

A recurrent phenotype that should be precociously identified on UPD(20)mat patients is the feeding difficulty in early infancy: these infants would not wake to eat, and would not cry to be fed. Five out of seven patients reported by Mulchandani et al. 2016 depended on direct gastric feeds in infancy [13].

5q35 microduplication and SHORT syndrome patients reported nowadays show increased recurrence of cardiac, hearing and ocular anomalies, so it’s mandatory to perform an early evaluation to guarantee appropriate follow up and care [17, 19].

In SHORT syndrome glucose blood levels monitoring is also important, although diabetes mellitus typically does not develop until early adulthood [17].

In conclusion, we confirm the usefulness and the suitability of NH-CSS as clinical diagnostic criteria for SRS, but we recommend excluding all other possible differential diagnoses before starting treatments. Our patients can be used as examples to delineate the correct clinical diagnostic approaches and to find some key points. Some differences may aid differentiating SRS from other aetiologies; relative macrocephaly is identified in our cohort only in the patient with *PIK3R1* mutation, while it is present in almost all patients with SRS. Hypotonia, developmental delay and intellectual disability are more frequent in patients with CNVs and in other imprinting disorders than SRS.

Lastly, we believe that patients scoring at least four of six criteria, according with the Consensus, could be diagnosed as clinical SRS if relative macrocephaly and protruding forehead is present, but some atypical features, as microcephaly or moderate/severe intellectual disability/neurodevelopmental delay, should not only questions the SRS diagnosis, but also suggests differential diagnosis.

## Data Availability

The data generated during the current study are not publicly available due to privacy or ethical restrictions.

## Abbreviations

BMI: Body mass index
CGH-Array: Array -Comparative Genomic Hybridization
CNV: Copy number variation
ECG: Electrocardiogram
FISH: Fluorescent in situ hybridization
GH: Growth Hormone
GHD: GH deficiency
IUGR: Intra-uterine growth restriction
MRI: Magnetic resonance imaging
NA: Not assessed
NH-CSS: Netchine–Harbison clinical scoring system
NGS: Next generation sequencing
SD: Standard Deviations
SGA: Small for gestational age
SNP: Single nucleotide polymorphism
SRS: Silver-Russell Syndrome
TS: Temple Syndrome
UD: Uniparental disomy
WES: Whole exome sequencing
